# Chemotherapy-associated oral complications in a south Indian population: a cross-sectional study

**DOI:** 10.25122/jml-2021-0342

**Published:** 2022-04

**Authors:** Suvranita Jena, Shamimul Hasan, Rajat Panigrahi, Pinali Das, Namrata Mishra, Shazina Saeed

**Affiliations:** 1. Department of Oral Medicine and Radiology, SCB Dental College and Hospital, Cuttack, India; 2. Department of Oral Medicine and Radiology, Faculty of Dentistry, Jamia Millia Islamia, New Delhi, India; 3. Department of Oral Medicine and Radiology, Institute of Dental Sciences, Siksha' O' Anusandhan University, Bhubaneswar, India; 4. Amity Institute of Public Health, Amity University, Noida, India

**Keywords:** cancer, chemotherapy, oral health physician, stomatotoxicity, quality of life, xerostomia

## Abstract

Over the years, chemotherapy (CT) has evolved as an essential therapeutic modality for cancer, with oral manifestations frequently encountered as complications of cancer CT. Our study aimed to assess the prevalence of oral complications during CT and evaluate the significance of independent risk factors (age, gender, socio-economic status, oral hygiene practices etc). A cross-sectional study was carried out in a tertiary cancer hospital in Bhubaneswar, Odisha, India, in which a total of 138 hospitalized patients undergoing CT and fulfilling the inclusion and exclusion criteria were included. Comprehensive history and rigorous clinical examination eliciting the oral manifestations were carried out. Around 60% of patients exhibited oral manifestations. Xerostomia and lichenoid reactions were the highest and lowest recorded manifestations. Higher frequencies of oral lesions occurred in patients with breast cancer, TNM stage III, and with the administration of the docetaxel. Also, patients in the older age group, poor socio-economic status, poor quality of life, poor oral hygiene practices, and longer CT duration demonstrated more oral lesions. Individuals subjected to a dental evaluation either before or during CT exhibited a reduction in the number of oral features. Several oral complications were reported in the present study. All patients undergoing chemotherapy must receive reinforcement of oral hygiene instructions and dental evaluation before, during, and after chemotherapy treatment. The study also emphasizes the importance of oral health physician inclusion in the multidisciplinary cancer treatment team.

## Introduction

The term cancer depicts an array of disorders entailing unrestrained cell growth. Cancer has evolved as a significant global public health threat in the recent past. Based on the World Health Organisation (WHO) estimation, cancer will result in 27 million incident cases, 17 million deaths, and 75 million people each year by 2030 [1].

A range of treatment strategies currently exists for cancer management: chemotherapy, radiotherapy, combination therapy, hormonal therapy, and immunotherapy [2]. Ever since the induction of folic acid antagonists and nitrogen mustards as chemotherapeutic agents for leukemias and lymphomas respectively, chemotherapy has shown remarkable advancements [3] and continues to be one of the most frequently employed present-day therapies for most cancer cases (>70% cases) [4].

The major drawback of chemotherapy is that it lacks selectivity, *i.e.*, it destroys or inhibits the growth of the cancerous cells and the rapidly multiplying normal cells, such as the bone marrow and oral mucosa [5]. Presently, the chemotherapeutic drugs may be either cytostatic or cytotoxic. Cytostatic drugs impair cancerous cell proliferation, whereas cytotoxic drugs result in cell destruction [6].

Published literature has documented that 40–60% of cancer-ridden patients are managed systemically. Among the CT-treated adult patients, nearly 40% elicit a varying degree of stomato-toxicities [5, 7], whereas 90% of children below 12 years manifest CT-induced oral lesions [1].

The gastrointestinal tract mucosal lining, including the oral mucosa, is extremely vulnerable to CT-induced stomato-toxicities. This site propensity occurs due to a plethora of risk factors, such as higher cellular turnover rates, distinct and heterogeneous microflora, and tissue trauma during normal oral functions [8].

CT-induced stomato-toxicities may influence the treatment protocols, possibly making it imperative to reduce the administered dose or even terminate the antineoplastic treatment, directly affecting patient survival [9]. The oral health physician should be able to diagnose various CT-induced oral manifestations and should ensure prompt management of these, thereby ameliorating the patient's oral and systemic health [10]. 

Several studies reporting the CT-induced stomato-toxicities in the pediatric population have been published [10–12], although there is still a scarcity of literature documenting the prevalence of CT-induced oral lesions in the adult population [4, 13].

With this background, our study aimed to assess the prevalence of oral manifestations during CT and evaluate the association of independent risk factors (age, gender, socio-economic status, oral hygiene practices etc) with the frequency of oral complications.

## Material and methods

A cross-sectional study was conducted for ten months (October 2020 and July 2021) in a tertiary cancer center in Bhubaneswar, Odisha. A total of 138 hospitalized patients undergoing CT, irrespective of gender, between 25–75 years of age, were included in the study. Newly diagnosed cancer cases, patients undergoing radiotherapy along with chemotherapy, patients with other systemic diseases (diabetes mellitus, cardiac, renal, and liver disorder) as systemic diseases are a common risk factor for oral complications, pregnant and lactating females, and unwilling patients were excluded from the study.

Patient's details, including name, age, gender, educational and socio-economic status were recorded. In addition, a detailed history, including the type of the carcinoma, type of chemotherapeutic drugs administered, TNM staging, number of chemotherapy cycles, duration of chemotherapy, and associated oral manifestation, were documented for all patients. Mucositis and viral infections were diagnosed by clinical appearance and oral symptoms. Objective evaluation of xerostomia was made when the tongue blade adhered to the oral mucosa. Candidiasis was diagnosed when candidal hyphae were demonstrated in smears fixed with alcohol and stained with the periodic acid Schiff (pas) method. Domain-specific scores were calculated manually by the researcher and data was transferred and coded on the excel sheet, and statistical analysis was done using Stata software, version 15.1.

## Results

A total of 138 patients were examined and assessed for various chemotherapy-related oral manifestations. Out of 138 patients, most patients were females (54.3%), and the rest were males (45.6%). 47.1% of participants were in the elderly age group above 61 years old. 23% were 51 to 60 years old, and the rest were younger than 50 years of age. Most of the participants in our cohort belonged to low (51.44%) and medium (33.33%) socio-economic strata. All participants had some level of education. 28% and 37.6% had primary and secondary education, while the rest were either graduates or post-graduates. We also evaluated the oral hygienic practices of the individuals, and most participants (44.2%) had poor oral hygiene practices (Table 1).

**Table 1.** Socio-demographic variables and their association with oral manifestations.

**Demographic Variables**	**N**	**%**	**Oral Manifestations(n)**		**p-value**
			**Yes**	**No**	
Age					
30–40	17	12.31884058	10	7	0.038006*
41–50	24	17.39130435	15	9	
51–60	32	23.1884058	26	6	
61 and above	65	47.10144928	55	10	
**Gender**					
Male	63	45.6	34	29	0.174372
Female	75	54.3	49	26	
**Socioeconomic status**					
Low	71	51.44927536	57	14	0.00001*
Medium	46	33.33333333	16	30	
High	21	15.2173913	10	11	
**Education level**					
Primary	39	28.26086957	21	18	0.961013
Secondary	52	37.68115942	27	25	
Graduation	23	16.66666667	12	11	
Post-graduation	24	17.39130435	14	10	
**Oral hygiene practices**					
Good	34	24.63768116	14	20	0.000063*
Fair	43	31.15942029	32	11	
Poor	61	44.20289855	51	10	

* – Statistically significant.

**Figure 1.**
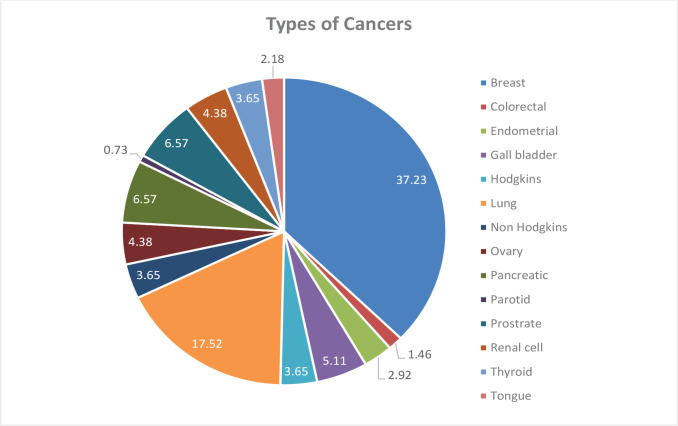
Different types of cancers recorded during chemotherapy.

Fourteen different types of carcinoma were recorded during the study. Among them, breast carcinoma was the most common recorded cancer (37.23%), followed by lung carcinoma (17.52%) and prostate and endometrial cancer (each 6.57%), whereas parotid carcinoma was the least encountered (0.73%) (Figure 1).

**Figure 2.**
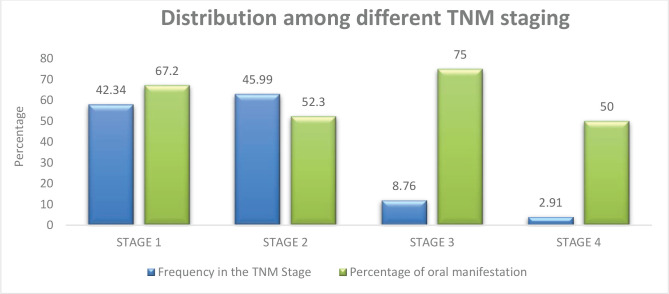
Patients in various TNM stages and the frequency of oral lesions in different stages.

Most of the study cohort were in stage 2 (45.99%), followed by those who were in stage 1 (42.34%). Very few patients belonged to stage 3 and 4, 8.76% and 2.91%, respectively. The cancer staging for all participants was based on the TNM classification followed globally (Figure 2).

**Figure 3.**
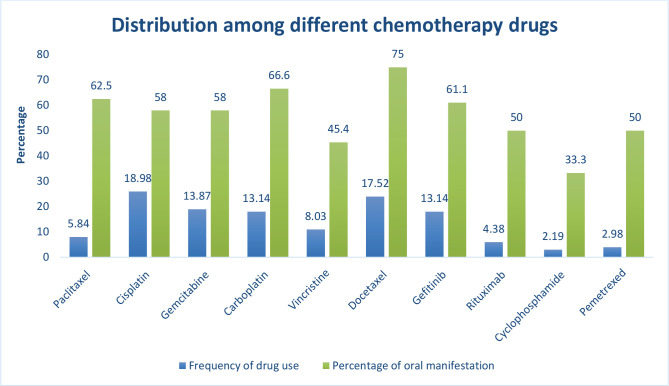
Oral manifestations among various chemotherapeutic drugs.

In our cohort, cisplatin was the most frequently administered chemotherapeutic drug, accounting for 18.98%, followed by docetaxel (17.52%), gemcitabine (13.87%), carboplatin (13.14%), gefitinib (13.14%), vincristine (8.03%), paclitaxel (5.84%), rituximab (4.38%), and pemetrexed (2.92%). Cyclophosphamide (2.19%) was the least frequently administered drug (Figure 3). 

**Figure 4.**
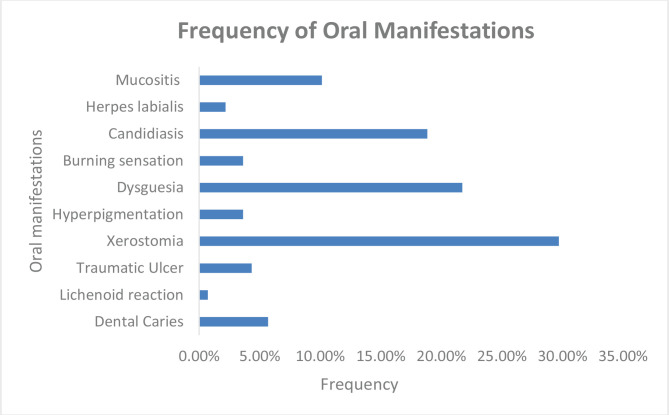
Frequency of various oral manifestations.

Around 60% of patients undergoing chemotherapy had at least one or more oral manifestations. The most common manifestation in our study was xerostomia (29.71%), and the least recorded was the lichenoid reaction (0.72%). Other manifestations like – dysgeusia (21.74%), candidiasis (18.84%), traumatic ulcer (4.35%), hyperpigmentation (3.62%), and burning sensation (3.62%) were also reported. Mucositis was reported in 10.14% of cases, out of which three patients (2.17%) had grade I mucositis, and the other eleven patients (7.97%) had grade II mucositis (Figure 4).

Oral manifestations were highest in the older age group (≥61 years), low-socio-economic status, and individuals with poor oral hygiene practices. The frequency of oral mucositis (OM) showed a significant association with age (p-value 0.038006); socio-economic status (p-value 0.00001); and oral hygiene practices (p-value 0.000063) (Table 1).

The number of oral manifestations was not associated with the number of CT cycles (p-value 0.684). However, it exhibited a significant association with the duration of the CT treatment (p-value 0.01243) and the quality of life of the individual during chemotherapy (p-value 0.0269), suggesting that the frequency of oral manifestations was higher in individuals with a longer duration of chemotherapy and those with poorer quality of life (Table 2).

**Table 2.** Association between oral manifestations, chemotherapy cycles, duration of chemotherapy and quality of life during chemotherapy, using the Kruskal-Wallis Test.

**Number of cycles**	**Number of oral manifestations (n)**						**p-value**
	**0**	**1**	**2**	**3**	**4**	**5**	
1 to 2	2	2	2	0	2	0	0.684
3 to 4	3	1	4	3	0	0	
5 to 6	1	2	6	3	2	0	
**Duration of CT (months)**	**Number of oral manifestations (n)**						**p-value**
	0	1	2	3	4	5	
<1 month	5	9	8	7	5	1	0.01243*
1–2 months	0	0	1	2	1	0	
≥3 months	2	1	4	3	0	0	
**Quality of Life during CT**	**Number of oral manifestations (n)**						**p-value**
	**0**	**1**	**2**	**3**	**4**	**5**	
Unaffected	2	3	1	0	0	0	0.0269*
Moderately affected	3	2	2	1	1	0	
Severely affected	2	3	4	6	7	2	

* – Statistically significant.

The number of oral manifestations showed significant association when dental evaluations were done before CT sessions (p-value 0.03078) and were highly significant among those evaluated during CT (p-value 0.00652), implying that a reduced number of oral complications were seen in individuals who had a dental evaluation done before and during CT (Table 3).

**Table 3.** Association between the frequency of oral manifestation and dental evaluations done before and during CT.

**Dental evaluation prior to CT**	**Number of oral manifestations (n)**						**p-value**
	**0**	**1**	**2**	**3**	**4**	**5**	
**Yes**	3	6	3	3	2	2	0.03078*
**No**	4	4	8	7	5	4	
**Dental evaluation during CT**	**Number of oral manifestations (n)**						**p-value**
	**0**	**1**	**2**	**3**	**4**	**5**	
**Yes**	0	1	0	3	0	0	0.00652*
**No**	4	7	9	7	6	3	

* – Statistically significant.

This study also assisted us in evaluating the relationship between TNM stages with the prevalence of oral manifestations. The prevalence of oral manifestations was the highest (75%) among individuals in stage 3, followed by those in stage 1 (67.2%) and stage 2 (52.3%) (Figure 2). 

The study delineated the frequency and distribution of oral manifestations among different chemotherapeutic drugs. Around 75% of patients under docetaxel showed oral manifestations, followed by carboplatin and paclitaxel, with a prevalence of 66.6% and 62.5%, respectively. Although cisplatin is the most frequently administered drug in our cohort, approximately 58% of the individuals experienced oral manifestations during chemotherapy, thus rendering it relatively safe (Figure 3). 

There was a significant association between the quality of life during CT and the number of oral manifestations (p-value 0.0269) (Table 2). Our study suggested that oral manifestations were higher in individuals with poorer quality of life.

## Discussion

Published literature reported that a varying degree of stomatotoxicities is elicited by nearly all the chemotherapy agents [1]. These oral complications may be acute or chronic and may arise during or after cancer chemotherapy. The commonly encountered CT-induced oral manifestations include mucositis, xerostomia, dysgeusia, salivary gland dysfunction, pain, and infections [3].

Since the emergence of chemotherapy in the last century, constant efforts have been taken to evaluate and ameliorate the effects of chemotherapeutic agents. This may be done either by dose intensification, or a combination of drugs, thereby alleviating their adverse effects [14].

Fourteen different types of carcinoma were recorded during the study. Breast carcinoma (37.23%), lung carcinoma (17.52%), and prostrate & endometrial cancer (each 6.25%) were the most common recorded carcinomas, whereas parotid carcinoma was the least encountered ca (0.73%). However, other studies highlighted a range of other carcinomas as the most commonly encountered, namely leukemias [1, 14–17], breast cancer [2, 7], lymphomas [4], and esophageal cancer [18]. 

Our study showed that cisplatin was the most prescribed chemotherapeutic drug (18.98%), followed by docetaxel (17.52%) and gemcitabine (13.87%), and the least prescribed drug was cyclophosphamide (2.19%). The most administered chemotherapeutic drugs in other studies were 5-fluorouracil [18], Aredia [19], 5-fluorouracil, cyclophosphamide, doxorubicin [7], and antimetabolites [20]. 

Most of the screened participants in our cohort were in stages I & II of TNM staging. Our findings contrast with other studies where most of the cohort were in advanced stages (stages II & III) [7]. 

Around 60% of patients undergoing chemotherapy had at least one or more oral manifestations. These results corroborate the findings from other studies [1, 4, 7, 14–21]. 

In our study, oral lesions were significantly associated with old age (p-value 0.038006). Similar findings were observed in other studies, where old age was a risk factor for the progression of oral lesions [14, 17]. Contrasting findings were reported in a study [22], which showed a higher frequency of oral lesions in younger individuals. However, no significant difference was observed in the frequency of oral lesions with age in other studies [16, 21].

The present study could not ascertain an association between gender and the frequency of oral manifestations. Similar results were observed in other studies [16, 23–27].

A less favored socio-economic status is generally associated with a higher incidence of oral disorders such as caries, *i.e.*, adverse oral conditions are highly susceptible to the development of oral complications during chemotherapy [28]. Oral manifestations were significantly associated with socio-economic status (p-value 0.00001) and were highest in patients with low socio-economic status. However, oral manifestations were seen irrespective of the patient's socio-economic status in another study [1]. 

The frequency of oral manifestations was highest in stage III, corresponding to the finding of another study [7]. The advanced clinical stages incorporate high dosages and an increased number of ct cycles, which generally will induce more toxic manifestations. 

The three most common manifestations were xerostomia (29.71%), followed by dysgeusia (21.74%), and candidiasis (18.84%), and the least recorded manifestation was the lichenoid reaction (0.73%). A similar prevalence of xerostomia was reported in other studies [1, 7, 21, 29]. However, other studies revealed either a lower prevalence of xerostomia [14, 18, 30], or a higher prevalence [4, 12, 18, 19, 31].

Few studies documented that alteration in chemical composition or physical properties of saliva, such as viscosity, may also impact the production of sensory signals perceived as dryness. Thus, alteration in viscosity could have also been an attributable factor for xerostomia. Patients with taste alterations may also complain of subjective oral dryness [32, 33]. This statement may contribute to the findings of our study.

Dysgeusia, delineated as a diminished or distorted taste ability, is a common oral complication encountered in almost 50–75% of cancer patients receiving CT, radiotherapy, or both [3]. As the chemotherapeutic agent may diffuse into the oral cavity, individuals may endure a displeasing metallic taste, generally a few weeks after chemotherapy initiation, returning to normal within a few weeks [34]. Dysgeusia was the 2^nd^ most common manifestation in our study (21.74%). Although, a higher prevalence of dysgeusia was reported in other studies [4, 6, 18, 31, 35]. Our study revealed that dysgeusia occurred primarily after the use of docetaxel, cisplatin, 5-fluorouracil, and cyclophosphamide. This finding agrees with the published literature [6]. 

During the progression of chemotherapy, myelosuppression may occur and may be associated with an increased prevalence of opportunistic bacterial, viral, and fungal infections [20]. Herpes simplex virus (HSV), varicella-zoster virus (VZV), and cytomegalovirus (CMV) accounts for most viral infections in patients subjected to CT [36].

A low prevalence of herpes labialis (2.17%) was reported in our study. A similar prevalence of herpetic lesions was reported in other studies [2, 18, 37], whereas other studies documented a slightly higher frequency [7, 14, 16] or a much higher frequency of herpetic lesions [21, 38]. Like other studies [21], the most common site of herpetic lesions was the dorsal tongue, hard palate, and gingiva. Herpetic lesions might have occurred due to reactivation of the latent virus resulting from the immunosuppression of the patients [10]. 

Fungal infections may be the primary factors for co-morbid states post-chemotherapy and may also enhance the likelihood of esophageal candidiasis [14]. Candida albicans is a commensal fungus of the upper and lower gastrointestinal tract involved with opportunistic mucosal and disseminated infections in immuno-suppressed individuals. The fungus can augment the proinflammatory epithelial reaction to the cytotoxic drug 5-fu *in vitro*, and it can facilitate a dysbiotic state in vivo, thereby causing a rapid oral mucosal barrier breach and resulting in life-threatening systemic dissemination [39].

Like other studies [14, 17, 21, 40], a moderate frequency of candidiasis (18.84%) was reported in our study. The study findings contrasted with the other reported studies, where a lower frequency of oral candidal infection was documented [1, 2, 7, 15, 16, 18, 23, 30, 31, 37]. Candidal prevalence in our study may be attributable to poor oral hygiene practices and the accompanying xerostomia.

Oral fungal infections may demonstrate various clinical types, although pseudomembranous or erythematous candidiasis are the most frequently encountered [41]. Our study demonstrated that pseudomembranous candidiasis was the most ordinary observed form, as per other reports [42–44], whereas erythematous candidiasis was the predominant variety in another study [21]. 

Oral mucositis (OM) is an iatrogenic complication of cancer chemotherapy, where there is inflammation and ulceration of the digestive tract mucosal lining. Mucositis has a site predilection for the non-keratinized mucosa (labial and buccal mucosa, floor of the mouth, the ventral tongue surfaces, and the soft palate) due to a rapid turnover rate and lack of a cornified layer [18, 21, 23, 45]. However, other studies reported that mucositis might occur in both keratinized and non-keratinized mucosa. The WHO provided a useful grading scale that combines objective and subjective elements (Table 4) [46].

**Table 4.** WHO Oral mucositis scale.

**Grade**	**Clinical presentation**
**0**	Normal
**1**	Soreness with/without erythema
**2**	Ulceration and erythema
**3**	Ulceration and extensive erythema, patient cannot swallow solid food
**4**	Mucositis of such severity that feeding is not possible

The prevalence of mucositis varies, even though the oral cavity is extremely vulnerable to the deleterious effects of chemotherapy. Severe mucositis occurs in bone marrow transplant patients (90%) and pediatric oncologic patients (65%), as they may be subjected to more contentious chemotherapy protocols. However, individuals subjected to chemotherapy for solid malignancies exhibit mild mucositis (21%) [21].

In our study, mucositis was reported in 10.14% of cases. These findings agreed with other studies [2, 37]. However, few other studies documented a moderate frequency [4, 15, 19–21], or a higher prevalence of oral mucositis [7, 12, 14, 16, 18, 28, 31, 47].

A relatively low prevalence of mucositis was reported in this study, presumably due to favorable oral health conditions before the chemotherapy induction, with a low percentage of oral diseases, which reduces the risk of developing oral manifestations during treatment [5]. Another attributable reason could be that in this study, individuals with various types of cancer were screened, which may, to some extent, be accountable for the low prevalence of mucositis.

Out of 14 patients with mucositis, three patients (2.17%) had grade I mucositis, and the other eleven patients (7.24%) had grade II mucositis. Similar findings were reported in other studies, where most of the patients exhibited grade I & II mucositis [2, 15]. However, another study revealed that 18% of patients exhibited features of severe grades of mucositis [47]. It is presumed that the prevalence and intensity of chemotherapy-induced mucositis partly occur due to a shift in the oral bacterial microflora. However, the association between periodontal pathogens and mucositis remains uncertain [48]. 

Published literature emphasized that chemotherapy has a negative impact on the oral health-related quality of life [49–51]. Xerostomia may cause speech difficulty resulting in oral tenderness and distress, and dysgeusia may cause appetite loss and result in malnutrition. These two conditions negatively impact the quality of life [4]. These features corroborate our study findings, where xerostomia and dysgeusia were the most frequently encountered oral manifestations, thus, accountable for the poor quality of life. Our study also revealed that patients with poor quality of life had a higher number of oral manifestations. 

The number of oral manifestations exhibited a positive association with the duration of the CT treatment. This finding agrees with the other study, in which the frequency and dose of the administered chemotherapeutic drugs correlated with the prevalence of adverse oral effects [4]. The authors emphasized that a prior meticulous oral evaluation is necessary for chemotherapy candidates. This enables oral physicians to diagnose and manage the impending focal infection. In addition, periodic surveillance of the patients undergoing chemotherapy should also be done throughout the scheduled treatment [31].

Our study revealed fewer oral manifestations in individuals who had a dental evaluation before and during CT. Reduced prevalence of oral complications was also reported in other studies, thus, highlighting the significance of dental assessment before chemotherapy initiation [7, 15]. However, another study did not show any association between oral manifestations and dental evaluation before or during CT [4]. Periodontal therapy effectively reduced plaque index, bleeding on probing and probing depth, and maintained attachment level in periodontitis cancer patients undergoing chemotherapy [52].

A higher prevalence of oral complications may accompany poor oral hygiene practices [20]. This fact highlights the importance of reinforcing oral health education programs, thus emphasizing the maintenance of oral hygiene and alleviating the deleterious stomatotoxic effects. Our study revealed a higher frequency of oral manifestations in individuals with poor oral hygiene practices. These findings corroborated with other study findings [16, 44]. However, contrasting results were seen in a study by Pels *et al.*, which revealed a higher prevalence of oral lesions in individuals with good oral hygiene [24]. A study by Ramirez *et al.* did not show any association between oral hygiene practices and the frequency of oral lesions [21].

Few studies also established that using 0.12% chlorhexidine gluconate mouthwash is beneficial in curbing oral complications post-CT [26, 53, 54].

Our study also delineated the frequency and distribution of oral manifestations among different chemotherapeutic drugs used. Around 75% of patients under docetaxel showed oral manifestations, followed by carboplatin and paclitaxel, with the prevalence of 66.6% and 62.5%, respectively. Although cisplatin is the most commonly used drug in our cohort, approximately 58% of the individuals experienced oral manifestations during chemotherapy, thus rendering it relatively safe.

Published literature demonstrated that stomatotoxic effects are frequent with conventional chemotherapeutic agents like antimetabolites (Fluorouracil, Xeloda), alkylating agents (cyclophosphamide, cisplatin), doxorubicin (Adriamycin), bleomycin, taxanes, and methotrexate [15, 16]. However, the studies failed to demonstrate any correlation between the prevalence of oral complications and the types of administered chemotherapeutic drugs [16, 23, 24, 29, 52].

The present study is a descriptive cross-sectional study, and the oral mucosal condition at the time of assessment might not depict the exact stomatotoxic chemotherapy effects. Hence, a longitudinal study enabling the periodic follow-up of the patients is generally preferred. This would delineate an extensive association between chemotherapy and its detrimental effects on oral health. This was a hospital-based study, so the results cannot be generalized to a larger population. Therefore, population-based studies with a larger sample size will be required. Also, at the time of the study, all the patients were in different chemotherapy phases, with a varying number of cycles, different treatment duration, and a range of administered chemotherapeutic drugs. All these factors may greatly influence the frequency of oral complications, and further studies should incorporate these factors.

## Conclusion

Among the reported oral complications in our study, xerostomia and lichenoid reactions were the most and least encountered manifestation, respectively. Patients in the older age group, poor socio-economic status, poor quality of life, poor oral hygiene practices, and longer CT duration reported a higher prevalence of oral lesions. Patients who had a dental evaluation done either before or during CT exhibited a reduction in the frequency of oral features. 

Although recent advances in chemotherapeutic treatment have considerably declined the mortality rates, the patients' miseries and grief continue. So, we as oral physicians should perform a pivotal role in managing cancer patients before, during, and after the chemotherapy session by enforcing a wide-ranging management approach.

## Acknowledgments

### Conflict of interest

The authors declare no conflict of interest.

### Ethical approval

This study was approved by the Institutional Ethics Committee (IEC), Institute of Medical Sciences, and Sum Hospital (dri/ims.su/soa 18/26/2020).

### Consent to participate

Informed consent was obtained from the participants, and their confidentiality was kept.

### Authorship

SJ contributed to the concept, design, manuscript writing, reviewing. SH contributed to concept, design, data analysis, manuscript writing. RP contributed to data collection, data analysis, statistical analysis, reviewing the manuscript. PD contributed to data collection and data analysis. NM contributed to data collection. SH contributed to concept, design, writing, reviewing and correspondence.
